# Supplementary figures and images for: Serum syndecan-1 concentration in hospitalized patients with heart failure may predict readmission-free survival
Source: PLoS One. 2021 Dec 8;16(12):e0260350. doi: 10.1371/journal.pone.0260350 (PMC8654157; doi:10.1371/journal.pone.0260350)

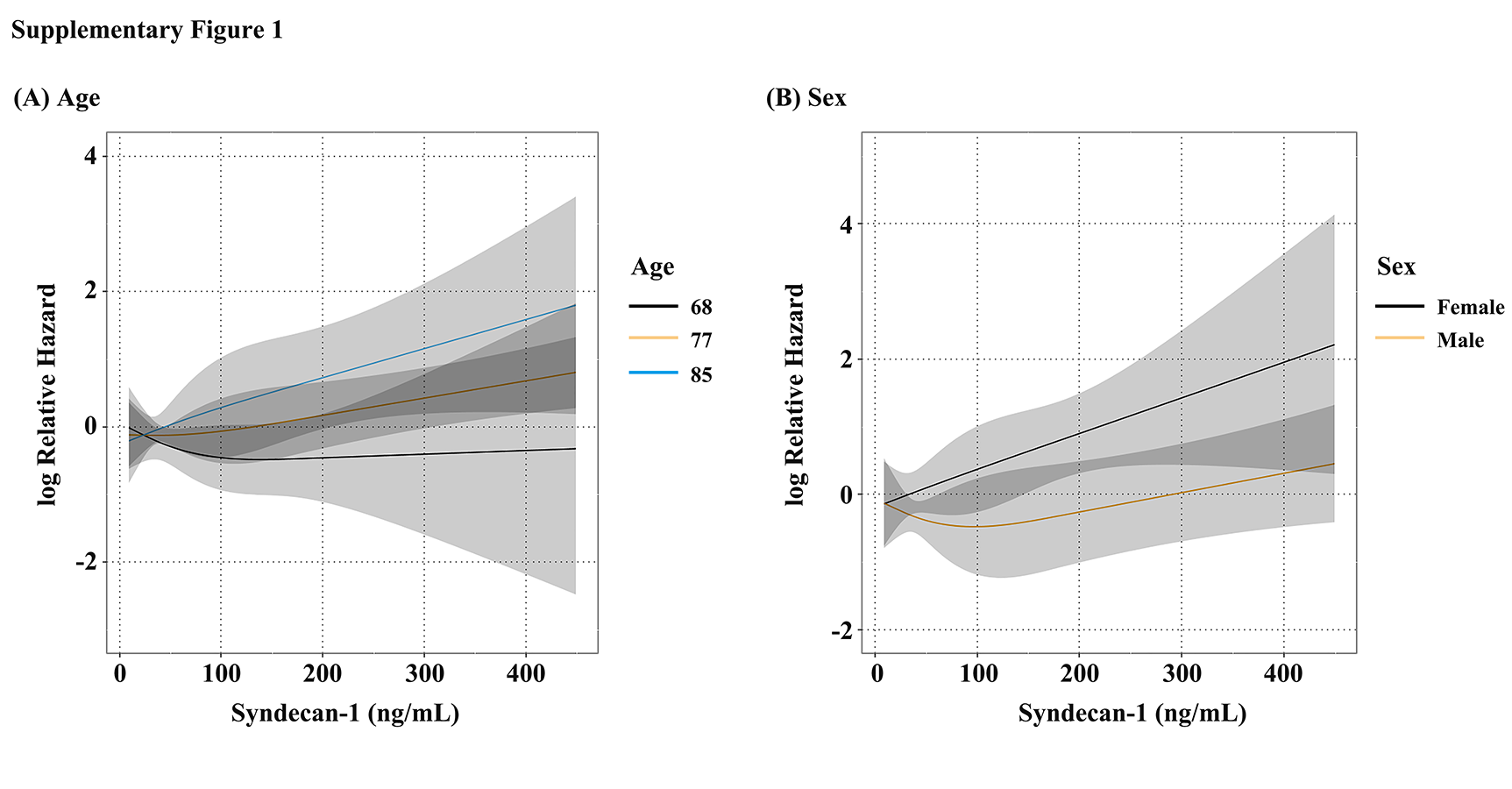

Supplement: S1 Fig — (TIF) [file pone.0260350.s001.tif]
