# Supplementary material for: Serum syndecan-1 concentration in hospitalized patients with heart failure may predict readmission-free survival
Source: PLoS One. 2021 Dec 8;16(12):e0260350. doi: 10.1371/journal.pone.0260350 (PMC8654157; doi:10.1371/journal.pone.0260350)
Supplement: S1 Table — (DOCX) [file pone.0260350.s002.docx]

**Supplementary Table 1**

| **Basal heart disease** | **N** | **Mean** | **SD** | **Median** | **Q1** | **Q3** |
| --- | --- | --- | --- | --- | --- | --- |
| Hypertensive heart disease | 26 | 42.2 | 27.8 | 33.5 | 23.8 | 48.3 |
| Ischemic heart disease (post-PCI) | 21 | 62.8 | 75.4 | 27.4 | 18.2 | 65.4 |
| Ischemic heart disease (post-CABG) | 12 | 57.1 | 82.2 | 30.1 | 25.7 | 38.3 |
| Ischemic heart disease (conservative treatment) | 7 | 112.6 | 164.5 | 45.5 | 38.0 | 49.4 |
| Arrhythmia (tachycardia) | 20 | 48.6 | 47.2 | 31.3 | 23.1 | 39.3 |
| Arrhythmia (bradycardia) | 1 | 59.7 | NA | 59.7 | 59.7 | 59.7 |
| Dilated cardiomyopathy | 17 | 59.0 | 57.7 | 42.5 | 32.0 | 61.0 |
| Hypertrophic cardiomyopathy | 5 | 52.9 | 27.3 | 55.3 | 41.6 | 66.7 |
| Other cardiomyopathy | 6 | 27.5 | 7.6 | 28.3 | 26.0 | 31.8 |
| Aortic valve stenosis (post-operation) | 6 | 95.4 | 96.8 | 56.6 | 38.2 | 120.6 |
| Aortic valve stenosis (conservative treatment) | 3 | 41.6 | 20.6 | 37.3 | 30.3 | 50.7 |
| Aortic valve insufficiency (post-operation) | 1 | 36.2 | NA | 36.2 | 36.2 | 36.2 |
| Aortic valve insufficiency (conservative treatment) | 5 | 29.1 | 6.3 | 28.4 | 26.6 | 30.9 |
| Mitral valve insufficiency (post-operation) | 3 | 213.7 | 319.5 | 34.1 | 29.3 | 308.3 |
| Mitral valve insufficiency (conservative treatment) | 8 | 384.8 | 854.3 | 45.3 | 28.7 | 52.6 |
| Tricuspid valve insufficiency (conservative treatment) | 3 | 80.5 | 86.1 | 49.4 | 31.8 | 113.6 |
| Congestive disease | 2 | 24.0 | 1.8 | 24.0 | 23.4 | 24.6 |
| Other | 6 | 48.2 | 37.8 | 30.6 | 26.5 | 61.1 |
