# Supplementary material for: Serum syndecan-1 concentration in hospitalized patients with heart failure may predict readmission-free survival
Source: PLoS One. 2021 Dec 8;16(12):e0260350. doi: 10.1371/journal.pone.0260350 (PMC8654157; doi:10.1371/journal.pone.0260350)
Supplement: S2 Table — (DOCX) [file pone.0260350.s003.docx]

**Supplementary Table 2**

| **Medication** | **Mean** | **SD** | **Median** | **Q1** | **Q3** |
| --- | --- | --- | --- | --- | --- |
| Beta-blocker | 71.5 | 217.7 | 31.1 | 22.5 | 51.5 |
| ACE inhibitor/ARB | 41.2 | 28.5 | 33.8 | 23.5 | 48.3 |
| Statin | 97.8 | 299.3 | 33.5 | 22.1 | 61.3 |
| Antiplatelets | 58.8 | 78.3 | 31.1 | 23.1 | 47.3 |
| Anticoagulants | 93.1 | 266.8 | 34.1 | 23.1 | 50.5 |
| Loop diuretics | 79.6 | 215.5 | 34.1 | 23.1 | 60.8 |
| Spironolactone | 81.5 | 243.4 | 34.2 | 23.2 | 55.6 |
| Tolvaptan | 79.4 | 102.3 | 46.6 | 27.1 | 72.6 |
| Catecholamine | 50 | 38.2 | 36.1 | 31.1 | 51.5 |
| Human atrial natriuretic peptide | 75.5 | 231.2 | 35.4 | 25.6 | 51.5 |
| Antibiotics | 69.9 | 102.4 | 34.2 | 28.2 | 54.2 |
