# Supplementary material for: Serum syndecan-1 concentration in hospitalized patients with heart failure may predict readmission-free survival
Source: PLoS One. 2021 Dec 8;16(12):e0260350. doi: 10.1371/journal.pone.0260350 (PMC8654157; doi:10.1371/journal.pone.0260350)
Supplement: S3 Table — (DOCX) [file pone.0260350.s004.docx]

**Supplementary Table 3: The association between BUN/creatinine and syndecan-1**

|  | **Coefficient** | **95% LCI** | **95% UCI** | **P value** |
| --- | --- | --- | --- | --- |
| Syndecan-1 (ng/mL)  37.04(median)-50 | 0.394 | 0.165 | 0.623 | 0.001 |
| Syndecan-1 (ng/mL)  37.04(median)-100 | 1.295 | 0.568 | 2.021 | <0.001 |
| Syndecan-1 (ng/mL)  37.04(median)-200 | 1.525 | 0.697 | 2.352 | <0.001 |
| Syndecan-1 (ng/mL)  37.04(median)-300 | 1.482 | 0.53 | 2.434 | 0.002 |
| Syndecan-1 (ng/mL)  37.04(median)-400 | 1.44 | 0.229 | 2.651 | 0.02 |
| Age (years) | 0.071 | 0.03 | 0.111 | 0.001 |
| Sex (male:female) | −3.941 | −4.886 | −2.995 | <0.001 |

CI, confidence interval
